# Cancer Mortality in People Treated with Antidepressants before Cancer Diagnosis: A Population Based Cohort Study

**DOI:** 10.1371/journal.pone.0138134

**Published:** 2015-09-14

**Authors:** Yuelian Sun, Peter Vedsted, Morten Fenger-Grøn, Chun Sen Wu, Bodil Hammer Bech, Jørn Olsen, Michael Eriksen Benros, Mogens Vestergaard

**Affiliations:** 1 Research Unit for General Practice, Department of Public Health, Aarhus University, Aarhus, Denmark; 2 Section for Epidemiology, Department of Public Health, Aarhus University, Aarhus, Denmark; 3 Section for General Practice, Department of Public Health, Aarhus University, Aarhus, Denmark; 4 Psychiatric Research Unit, Copenhagen University Hospital, Copenhagen, Denmark; University of Alabama at Birmingham, UNITED STATES

## Abstract

**Background:**

Depression is common after a cancer diagnosis and is associated with an increased mortality, but it is unclear whether depression occurring before the cancer diagnosis affects cancer mortality. We aimed to study cancer mortality of people treated with antidepressants before cancer diagnosis.

**Methods and Findings:**

We conducted a population based cohort study of all adults diagnosed with cancer between January 2003 and December 2010 in Denmark (N = 201,662). We obtained information on cancer from the Danish Cancer Registry, on the day of death from the Danish Civil Registry, and on redeemed antidepressants from the Danish National Prescription Registry. Current users of antidepressants were defined as those who redeemed the latest prescription of antidepressant 0–4 months before cancer diagnosis (irrespective of earlier prescriptions), and former users as those who redeemed the latest prescription five or more months before cancer diagnosis. We estimated an all-cause one-year mortality rate ratio (MRR) and a conditional five-year MRR for patients who survived the first year after cancer diagnosis and confidence interval (CI) using a Cox proportional hazards regression model. Overall, 33,111 (16.4%) patients redeemed at least one antidepressant prescription in the three years before cancer diagnosis of whom 21,851 (10.8%) were current users at the time of cancer diagnosis. Current antidepressant users had a 32% higher one-year mortality (MRR = 1.32, 95% CI: 1.29–1.35) and a 22% higher conditional five-year mortality (MRR = 1.22, 95% CI: 1.17–1.26) if patients survived the first year after the cancer diagnosis than patients not redeeming antidepressants. The one-year mortality was particularly high for patients who initiated antidepressant treatment within four months before cancer diagnosis (MRR = 1.54, 95% CI: 1.47–1.61). Former users had no increased cancer mortality.

**Conclusions:**

Initiation of antidepressive treatment prior to cancer diagnosis is common and is associated with an increased mortality.

## Introduction

Depression is common after cancer and has a major impact on the quality and the duration of life [1,2]. However, it has not been well-studied to which extent depression occurring before a cancer diagnosis affects cancer survival although some plausible mechanisms indicate that this could be so. Firstly, a diagnostic delay of cancer may occur in people with depression as they may be less likely to attend screening programs or seek care [3,4] or their physical complaints may be masked by their underlying mental condition [5–7]. Secondly, an erroneous diagnosis of depression may be given to people with non-specific symptoms of undetected cancer mimicking those of depression such as fatigue, weight loss, and sleep disturbances [8,9] or to people whose cancer present itself with psychiatric symptoms due to a direct effect from a brain tumor or a paraneoplastic syndrome, for instance [10,11]. Misinterpretations of early cancer symptoms may cause a diagnostic delay and a poorer prognosis if the patient is being treated for depression instead of being examined for cancer. Finally, a suboptimal treatment of the cancer may occur in people with depression as they may be less likely to follow treatment and rehabilitation regimens or if the antidepressant treatment interacts with the cancer treatment [12–15]. A few studies have shown that people treated for psychiatric diseases within secondary health care have an increased cancer mortality [13,16–20], but it is unclear whether this association also apply to the much more prevalent type of depression treated in primary care.

We conducted a nationwide study to estimate the cancer mortality of people who were treated with antidepressants in the Danish healthcare system before their cancer diagnosis.

## Methods

### Study design and study population

We conducted a cohort study of adults (> = 18 years old) diagnosed with any incident cancer (except non-melanoma skin cancer) between 1 January 2003 and 31 December 2010 in Denmark (n = 215,826). We obtained cancer diagnoses from the Danish Cancer Registry [21], which contains data on all cancer cases since 1943; and we linked the study population to other nationwide registers using a unique personal identification number given to all Danish citizens. We excluded people who were not living in Denmark in the three years before cancer diagnosis (n = 1,761), who had a missing value for sex (n = 1,498) or for marital status at the time of diagnosis (n = 9,568), which left 201,662 people for the study.

### Assessment of cancer diagnosis

In the Danish Cancer Registry [21], cancer diagnoses were coded according to a modified Danish version of the International Classification of Diseases, 7^th^ revision (ICD-7) from 1943 to 1977 and the ICD for Oncology (ICD-O) afterwards; since 1978 onwards, the codes have been converted into the ICD-10 codes. All people in the study population with a first time cancer diagnosis during the study period were identified using the ICD-10 codes of C00-C43 and C45-C97. All people with previous cancers diagnoses, except for non-melanoma skin cancer, were excluded. The date of diagnosis was based on the Danish Cancer Registry’s definition, which is the first day in the admission where the diagnosis was confirmed.

Cancer stage at diagnosis was categorized into local, regional, distant, and unknown based on the anatomic extent of the tumor, including tumor size (T), the number of lymph nodes involved (N), and the presence of metastases (M). The “unknown” category include tumors of high T class, which are known to carry a considerable risk of lymph node or distant metastasis, but it often lacked information of either one or both. We used a verified algorithm to categorize the stage at the time of diagnosis for lung, colorectal, breast, melanoma, prostate, and bladder cancers [22–27]. The algorithms were developed for each type of cancer according to the available codes for T, N, and M but missing data were allowed if the available information provided sufficient and clinically meaningful information to categorize cases. For all other cancer types, we used an algorithm originally developed for colorectal cancer to categorize the stage [27].

### Assessment of depression

Information on antidepressants was obtained from the Danish National Prescription Registry [28]. The registry contains prescriptions of medicines dispensed at any Danish pharmacy since 1995. In the register, drugs are classified according to the anatomical therapeutical chemical (ATC) system [28]. Antidepressants were identified using the code of N06A. We included the following groups of antidepressants: selective serotonin reuptake inhibitors (N06AB), tricyclic antidepressants (N06AA), and other antidepressants including serotonin-norepinephrine reuptake inhibitors and noradrenergic and specific serotonergic antidepressants (N06AX16, N06AX21, N06AX03, N06AX11). The prescription register data contain information on the prescription issue date, name and code of a medicine, and total number of pills prescribed. Unfortunately, no information was available in the register on the prescribed daily dose.

Antidepressant treatment initiation was defined as the day of the first prescription of the latest antidepressant treatment episode. Two prescriptions redeemed within a 12-month period were regarded as part of the same antidepressant treatment episode. People who redeemed the last antidepressant prescription within four months of the cancer diagnosis were categorized as current users, and people who redeemed the last antidepressant prescription five or more months before the cancer diagnosis were categorized as former users.

### Vital status

Information on death or emigration was obtained from the Danish Civil Register, which was established in 1968 and covers all people with a permanent address in Denmark [29].

### Potential confounders

Information on sex, birth date, and marital status was obtained from the Danish Civil Register [29], and information on education from the Integrated Database for Labour Market Research, which was established in 1980 and which contains data on occupational status, income, and education on all individuals with a Danish citizenship. In the study population, 14,739 (7%) had a missing value on education at the time of cancer diagnosis, and they were categorized in the lower education group because 79% of them were above the age of 89 years; and for that age group, only elementary school education was common. Information on comorbidity was obtained from the Danish Hospital Register which contains diagnosis on outpatients and inpatients from all somatic hospitals in Denmark [30]. We calculated a Charlson comorbidity index [31], by summarizing disease points for 15 non-cancer health conditions in the 10 years before cancer diagnosis (1 point for acute myocardial infarction, congestive heart failure, peripheral vascular disease, cerebrovascular disease, dementia, chronic pulmonary disease, rheumatologic disease, peptic ulcer disease, mild liver disease, and diabetes without complications; 2 points for diabetes with chronic complications, hemiplegia or paraplegia, and renal disease; 3 points for moderate or severe liver disease; and 6 points for AIDS/HIV).

### Statistical analyses

We estimated an all-cause one-year mortality rate ratio (MRR) for all participants and a conditional five-year MRR for patients who survived the first year after cancer diagnosis for current antidepressant users and for former antidepressant users. Cancer patients who had not been prescribed antidepressants in the three years before cancer diagnosis were used as a reference group. We used Cox proportional hazards regression model and cancer patients were followed from the day of cancer diagnosis until death, emigration, the end of five years of follow-up, or 31 December 2010, whichever came first. All the analyses were performed with STATA 12 (Stata Corp, College Station, TX).

We modelled the impact of initiation time of an antidepressant treatment using right restricted cubic splines with four knots (120, 240, 480, 720 days). We used the initiation timing in days (1–720 days) for people who initiated an antidepressant treatment in the two years before cancer diagnosis and set a value of 750 days (25 months) for people whose earliest prescription of an antidepressant treatment was in the 3rd year before cancer diagnosis or earlier. We added a parameter to the model allowing people treated with antidepressants to be different from people who were not, regardless of the time of initiation. Our findings from the cubic spline models therefore represent results relative to the reference group. In supplementary analyses, we modeled the association using timing of antidepressant treatment initiation before cancer diagnosis as a categorical variable (0–4, 5–8, 9–12, 13–16, 17–20, 20–24, 25+ months).

In the Cox regression model, we adjusted for sex, age at time of diagnosis (five-year interval), cancer stage at the time of diagnosis (local, regional, distant, unknown), Charlson comorbidity index (0, 1–2, 3+), marital status at the time of diagnosis (married or cohabiting, living alone), education (lower, medium, higher) at the time of diagnosis, and calendar year (2003–2004, 2005–2007, 2008–2010). Types of cancer were treated as strata in the analysis where cancers were grouped according to organ system.

Additionally, we estimated specific MMRs according to genders, types of cancers (lung, breast, prostate, bladder, colorectal cancer, and brain), types of antidepressants (selective serotonin reuptake inhibitors, tricyclic antidepressants, or others), and cancer stages (localized, regional, distant, and unknown).

## Results

Among the 201,662 cancer patients included in the analyses, a total of 33,111 (16.4%) had redeemed at least one prescription for antidepressants during the three years before the cancer diagnosis. Of these, 21,851 (10.8%) were current antidepressant users and 11,260 (5.6%) were former antidepressant users. Among current users, the antidepressant treatment was initiated 0–4 months (N = 4,304; 19.7%), 5–24 month (N = 10,220; 46.8%) and 24+ months (N = 7,327; 33.5%) before cancer diagnosis, respectively. Current antidepressant users were more likely to be female, older, and living alone and to have a shorter education, a higher score on the Charlson comorbidity index, and an advanced cancer stage than patients who did not use antidepressants within three years before the cancer diagnosis (Table 1). People with breast cancer, prostate cancer, or colorectal cancer–but not people with bladder cancer and lung cancer—were more likely to have an advanced stage at the time of cancer diagnosis if they initiated an antidepressant treatment 0–4 months before cancer diagnosis compared with the reference group (S1 Table).

**Table 1:** Characteristics of the study population according to antidepressant (AD) treatment in the three years before cancer diagnosis.

	AD in the 3 years before cancer diagnosis							
Characteristics	No		Yes (n = 33,111)					
	(n = 168,551)		Current AD user ^a^ at time of cancer diagnosis (n = 21,851)				Former AD user ^b^ (n = 11,260)	
			AD treatment initiated within 4 months of the cancer diagnosis (n = 4,304)		AD treatment initiated 5+ months before cancer diagnosis (n = 17,547)			
	No.	%	No.	%	No.	%	No.	%
Sex								
Male	87,359	51.8	2,132	49.5	6,309	36.0	4,614	41.0
Female	81,192	48.2	2,172	50.5	11,238	64.0	6,646	59.0
Age (years) at time of cancer diagnosis								
18–39	6,695	4.0	86	2.0	303	1.7	361	3.2
40–49	11,656	6.9	210	4.9	860	4.9	832	7.4
50–59	27,743	16.5	609	14.1	2,608	14.9	2,108	18.7
60–69	50,712	30.1	1,114	25.9	4,542	25.9	3,218	28.6
70–79	45,370	26.9	1,304	30.3	5,017	28.6	2,870	25.5
80–89	26,375	15.6	981	22.8	4,217	24.0	1,871	16.6
Marital status at time of cancer diagnosis								
Married or cohabiting	104,608	62.1	2,301	53.5	8,447	48.1	5,771	51.3
Living alone	63,943	37.9	2,003	46.5	9,100	51.9	5,489	48.7
Education at time of cancer diagnosis								
High	31,234	18.5	594	13.8	2,470	14.1	1,819	16.2
Medium	61,216	36.3	1,414	32.9	5,457	31.1	3,907	34.7
Low	76,101	45.2	2,296	53.3	9,620	54.8	5,534	49.1
Year at time of cancer diagnosis								
2003–2004	38,312	22.7	987	22.9	3,671	20.9	2,570	22.8
2005–2007	61,827	36.7	1,757	40.8	6,349	36.2	4,151	36.9
2008–2010	68,412	40.6	1,560	36.2	7,527	42.9	4,539	40.3
Charlson comorbidity index								
0-	121,016	71.8	2,361	54.9	8,790	50.1	6,512	57.8
1–2	39,122	23.2	1,494	34.7	6,604	37.6	3,653	32.4
3+	8,413	5.0	449	10.4	2,153	12.3	1,095	9.7
Cancer stage at time of diagnosis								
Local	59,170	35.1	978	22.7	5,509	31.4	3,913	34.8
Regional	24,708	14.7	510	11.8	2,560	14.6	1,663	14.8
Distant	29,507	17.5	1,109	25.8	3,317	18.9	2,015	17.9
Unknown or missing	55,166	32.7	1,707	39.7	6,161	35.1	3,669	32.6
Types of cancer								
Head and neck cancers	5,999	3.6	120	2.8	704	4.0	448	4.0
Upper digestive organs	5,574	3.3	145	3.4	615	3.5	360	3.2
Lower digestive organs	23,920	14.2	435	10.1	2,326	13.3	1,360	12.1
Liver and pancreas	7,209	4.3	301	7.0	832	4.7	564	5.0
Lung cancer	21,077	12.5	998	23.2	2,976	17.0	1,823	16.2
Sarcoma	2,084	1.2	58	1.3	154	0.9	133	1.2
Malignant melanoma	9,124	5.4	113	2.6	687	3.9	484	4.3
Breast cancer	26,663	15.8	452	10.5	3,272	18.7	2,048	18.2
Female genital organs	9,501	5.6	159	3.7	1,051	6.0	676	6.0
Male genital organs	26,666	15.8	443	10.3	1,614	9.2	1,289	11.4
Kidney and bladder	8,820	5.2	231	5.4	843	4.8	528	4.7
Brain	2,405	1.4	223	5.2	248	1.4	128	1.1
Secondary and unspecified sites	4,988	3.0	245	5.7	766	4.4	432	3.8
Lympatic and haematopoietic tissues	11,493	6.8	316	7.3	1,107	6.3	761	6.8
Others	3,010	1.8	63	1.5	349	2.0	226	2.0

^a^: Current AD users refers to cancer patients who redeemed an AD prescription within 4 months before the cancer diagnosis.

^b^: Former AD users refers to cancer patients who redeemed the last AD prescription more than 4 months before the cancer diagnosis.

**Fig 1:**
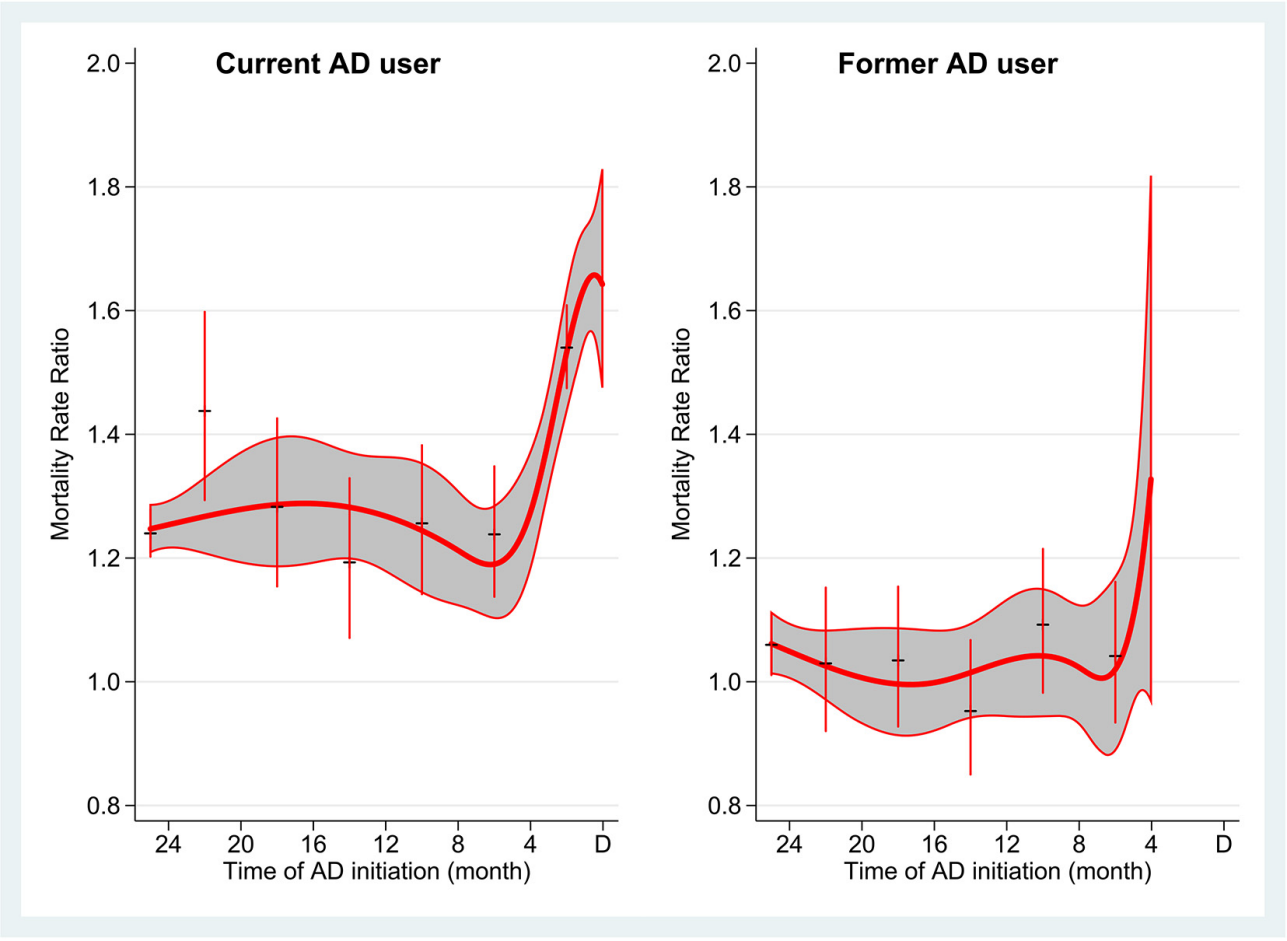
Mortality rate ratio^a^ in the first year after cancer diagnosis for current antidepressant (AD) users^b^ and former AD users^c^ according to the time of AD treatment initiation before cancer diagnosis (indicated by D). A bold curve and the 95% confidence band indicated by the grey area are results from Cox regression analyses by modelling the association of time of AD initiation using right restricted cubic splines and spike estimates are results by categorizing time of AD initiation into groups. ^a^ Adjusted for sex, age at time of diagnosis, stage of cancer, Charlson comorbidity index, marital status, education, and calendar year. ^b^ Current AD users refers to cancer patients who redeemed an AD prescription within 4 months before the cancer diagnosis. ^c^ Former AD users refers to cancer patients who redeemed the last AD prescription more than 4 months before the cancer diagnosis.

Current antidepressant users had a higher one-year mortality (MRR: 1.32; 95% CI: 1.29–1.35) and a higher conditional five-year mortality (MRR: 1.22, 95% CI: 1.17–1.26) than the reference group (Table 2). The estimates did not change much when we adjusted for stage of cancer in the model (Table 2). Current users had a particularly high mortality during the first year after cancer diagnosis, if they had initiated the antidepressant treatment during the four months before cancer diagnosis (average MRR 1.54 (95% CI: 1.47–1.61); Fig 1).

**Table 2:** Mortality rate ratio (MRR) of cancer mortality after cancer diagnosis for current antidepressant (AD) user ^a^ and former AD user ^b^ at time of cancer diagnosis.

AD treatment before diagnosis	Population	Person years (PYs)	No. of deaths	Mortality/ 100 PYs	Crude MRR	Adjusted ^c^ MRR (95%CI)	Adjusted ^d^ MRR (95%CI)
1-year mortality							
No ^e^	168,551	130,809	43,631	33.4	Ref	Ref	Ref
Current user ^a^	4,304	2,539	2,138	84.2	1.75	1.31 (1.28; 1.34)	1.32 (1.29; 1.35)
Former user ^b^	2,331	1,610	872	54.2	1.21	1.05 (1.01; 1.08)	1.07 (1.03; 1.11)
Conditional 5-year mortality if patients survived the first year after cancer diagnosis							
No ^e^	105,458	236,283	23,799	10.1	Ref	Ref	Ref
Current user ^a^	1,783	3,735	598	16.0	1.40	1.19 (1.15; 1.24)	1.22 (1.17; 1.26)
Former user ^b^	1,226	2,601	386	14.8	1.10	1.02 (0.97; 1.08)	1.05 (1.00; 1.11)

^a^ Current AD users refers to cancer patients who redeemed an AD prescription within 4 months before the cancer diagnosis.

^b^ Former AD users refers to cancer patients who redeemed the last AD prescription more than 4 months before the cancer diagnosis.

^c^ Adjusted for sex, age at time of diagnosis (5-year interval), Charlson comorbidity index (0, 1–2, 3+), marital status (married or cohabiting, living alone), education (low, medium, high), and calendar year (2003–2004, 2005–2007, 2008–2010).

^d^ Adjusted for cancer stage at time of diagnosis (local, regional, distant, and unknown) besides the factors included in the analysis.

^e^ The reference refers to cancer patients who had no AD prescription in the 3 years before cancer diagnosis.

Former antidepressant users had a 7% higher mortality (MRR: 1.07, 95% CI: 1.03–1.11) during the first year and a 5% higher conditional five-year mortality (MRR: 1.05, 95% CI: 1.00–1.11) (Table 2). This association became less clear when we did the analyses according to the time of initiation of antidepressant treatment for the former antidepressant users (Fig 1, S1 Fig).

**Fig 2:**
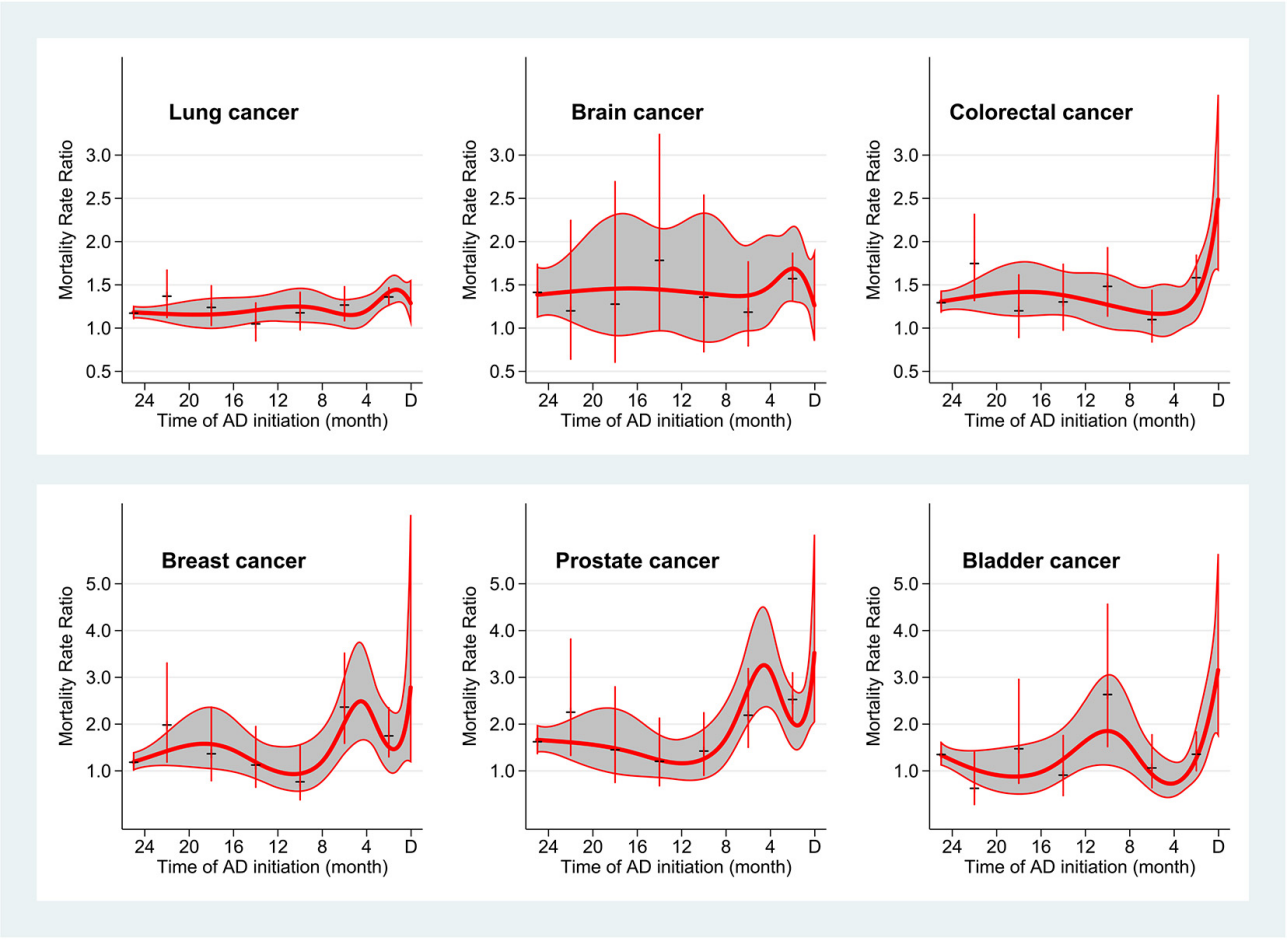
Mortality rate ratio in the first year after cancer diagnosis according to the time of antidepressant (AD) treatment initiation before cancer diagnosis for current AD users diagnosed with different types of cancer.

The results of the analyses where timing of antidepressant treatment initiation before cancer diagnosis was used as a categorical variable were similar to the results of the analyses using cubic spline. The estimates did not vary much in relation to the type of cancer (Fig 2), type of antidepressant medication (S2 Fig), sex of patients (S3 Fig), and stage of cancer at the time of diagnosis (S4 Fig).

## Discussion

This large population-based cohort study showed that one out of ten cancer patients were treated with antidepressants at the time of the cancer diagnosis and they had a significantly higher mortality, in particularly if the treatment was initiated shortly before the cancer diagnosis. The association did not apply to former antidepressant users and was not strongly altered by type of antidepressant or cancer type.

Previous studies have shown that people treated for depression in secondary health care had a 30–60% higher mortality after cancer diagnosis compared to those with no such history [13,16–20]. Our findings show that the association also applies to the much larger group of people treated for depression in primary care. One study found that the increased mortality was primarily found shortly after the cancer diagnosis [17], whereas our results indicate that the risk may be high for at least five years. Several studies have found depressive symptoms or clinically depressive episodes after a cancer diagnosis are associated with a higher cancer mortality [13,16–20,32,33], but our study indicates that this also applies to people who initiate treatment with antidepressant prior to their cancer diagnosis.

Our study has several strengths including a large cohort consisting of all people diagnosed with cancer during an eight-year period in Denmark, virtually complete follow-up, and valid and complete information on antidepressant prescription, cancer diagnosis, and mortality. We obtained information on antidepressant drugs from the Danish National Prescription Registry [28], which holds complete information on all prescriptions redeemed at any Danish pharmacy. Antidepressants are mainly used for depression, especially in middle-aged and older people, although they are also used for other conditions such as anxiety and pain. Information on cancer diagnoses was obtained from the Danish Cancer Registry [21]. The proportion of microscopically-confirmed cancers in the register has been about 93% since 1978, and the proportion of histologically-confirmed cancers was about 80% during 1988–1992 [21]. The completeness of cancer registration is high (98%) for non-skin cancers [21]. The cancer stage reflects the severity and the spread of the cancer and is strongly related to the cancer prognosis. There is substantial variation in the register in relation to the completeness of cancer stage according to the tumor size, the lymph nodes involved, and the presence of metastases. Complete information on cancer stage was available for 86% of bladder cancer, 82% of lung cancer, and 80% of melanoma, while the figure was only 66% for breast cancer and prostate cancer [34]. People with cancer with no information on stage tended to be older and have a higher level of comorbidity [34]. Information on vital status, which was used for calculation of mortality is updated daily with validity close to 100%.

The estimates did not change much after adjusting for several important confounding factors such as educational level, marital status, and comorbidity. However, we cannot exclude that residual confounding by factors not accounted for may play a role such as life style factors.

Early cancer diagnosis may be more difficult in people with depression because they tend to be less likely to attend cancer screening programs, and to be less likely to seek medical help for physical symptoms beyond depression [4,35]. Furthermore, an erroneous diagnosis of depression may be given to people with non-specific symptoms of undetected cancer mimicking those of depression such as fatigue, weight loss, and sleep disturbances [8,9] or to people whose cancer present itself with psychiatric symptoms due to a direct effect from a brain tumor or a paraneoplastic syndrome, for instance. We have previously shown that during the first months after a first-time psychiatric contact, people were more likely diagnosed with a cancer, especially brain cancer and small cell lung cancer [36]. If people with early cancer symptoms are treated for depression instead of being examined for cancer it may lead to a diagnostic delay that may cause a more advanced cancer stage at the time of diagnosis, just as we found. However, the association between depression and cancer mortality did not disappear when adjusting for cancer stage, which indicates that the excess mortality is not explained entirely by delayed diagnosis. However, residual confounding due to incomplete information on stage cannot be ruled out.

Depression may also have an independent effect on mortality among cancer patients. Psychological stress involves neuroendocrine response, immune response, or angiogenesis, which may affect cancer progression [14,15]. Our study as well as other studies [20,37] found that antidepressant treatment at the time of diagnosis was associated with higher mortality among women with breast cancer. This is noteworthy as breast cancer is diagnosed often basing on a lump in the breast, which is unlikely to be taken for depression. This suggests that the poorer mortality could not be explained by a misdiagnosis of undetected cancer as psychiatric distress.

The time interval from patient referral to cancer diagnosing to diagnosis confirmation is a major emotional strain for many patients. Some people may develop a depression and initiate treatment with antidepressants. A higher mortality is expected among people who are prescribed antidepressants immediately before the cancer diagnosis if people with the more severe cancers are more likely to develop depression during the waiting time for a diagnosis.

Poor cancer treatment compliance and poor engagement in rehabilitation programs among patients with depression may be another explanation. Studies have shown that people with depression or depressive symptoms are less likely to access medical services including surgery and radiotherapy, and they have fewer chemotherapy sessions [12,13]. A recently published study indicates that men with depressive disorder before prostate cancer diagnosis are less likely to get definitive cancer therapy like surgery and radiotherapy although they are more likely to receive expectant management defined as watchful waiting or active surveillance [38]. Previous studies have showed that the association remains even after adjusting for factors related to the stage of cancer and treatment of cancer [20,37], which indicates that other mechanisms may play a role. Antidepressant treatment could theoretically have an independent effect on mortality for example by interacting with cancer treatment. A recent study indicated that tricyclic antidepressant improved the survival from small cell lung cancer [38] and we found that the association effect did not depend on the type of antidepressant.

In conclusion, the ten percent of cancer patients who used antidepressants at the time of diagnosis constitutes a vulnerable group of patients with an increased mortality and a need for health care. Mounting evidence indicate that collaborative care models are effective in treating depression comorbid with medical conditions including cancer [39,40]. In these models, care managers, psychiatrists, and medical specialists collaborate with the patient’s primary care physician to provide systematic, and proactive treatment and follow-up.

## Supporting Information

S1 Fig Conditional five-year mortality rate ratio after cancer diagnosis for current antidepressant (AD) users and former AD users who survived in the first year according to the time of AD treatment initiation before cancer diagnosis (indicated by D). (EPS)

S2 Fig Mortality rate ratio in the first year after cancer diagnosis according to the time of antidepressant (AD) initiation before cancer diagnosis for current AD users who were prescribed with different types of AD. (EPS)

S3 Fig Mortality rate ratio in the first year after cancer diagnosis according to the time of antidepressant (AD) initiation before cancer diagnosis for current AD users among men and women. (EPS)

S4 Fig Mortality rate ratio in the first year after cancer diagnosis according to the time of antidepressant (AD) initiation before cancer diagnosis for current AD users who were at a localized stage and an advanced stage at time of cancer diagnosis. (EPS)

S1 Table Stage of cancer at time of diagnosis according to antidepressant (AD) treatment in the three years before cancer diagnosis. (DOCX)
