# Correction: Cancer Mortality in People Treated with Antidepressants before Cancer Diagnosis: a Population Based Cohort Study

**DOI:** 10.1371/journal.pone.0155453

**Published:** 2016-05-06

**Authors:** Yuelian Sun, Peter Vedsted, Morten Fenger-Grøn, Chun Sen Wu, Bodil Hammer Bech, Jørn Olsen, Michael Eriksen Benros, Mogens Vestergaard

In Table 2, the numbers of Population, Person years (PYs), No. of deaths, and Mortality/ 100 PYs are listed incorrectly for current user and former user. Please see the corrected Table 2.

**Table 2:** Mortality rate ratio (MRR) of cancer mortality after cancer diagnosis for current antidepressant (AD) user ^a^ and former AD user ^b^ at time of cancer diagnosis.

AD treatment before diagnosis	Population	Person years (PYs)	No. of deaths	Mortality/ 100 PYs	Crude MRR	Adjusted ^c^ MRR (95%CI)	Adjusted ^d^ MRR (95%CI)
1-year mortality							
No ^e^	168,551	130,809	43,631	33.4	Ref	Ref	Ref
Current user ^a^	21,851	14,503	8,744	60.3	1.75	1.31 (1.28; 1.34)	1.32 (1.29; 1.35)
Former user ^b^	11,260	8,374	3,396	40.6	1.21	1.05 (1.01; 1.08)	1.07 (1.03; 1.11)
Conditional 5-year mortality if patients survived the first year after cancer diagnosis							
No ^e^	105,458	236,283	23,799	10.1	Ref	Ref	Ref
Current user ^a^	10,767	22,131	3,172	14.3	1.40	1.19 (1.15; 1.24)	1.22 (1.17; 1.26)
Former user ^b^	6,610	14,646	1,621	11.1	1.10	1.02 (0.97; 1.08)	1.05 (1.00; 1.11)

^a^ Current AD users refers to cancer patients who redeemed an AD prescription within 4 months before the cancer diagnosis.

^b^ Former AD users refers to cancer patients who redeemed the last AD prescription more than 4 months before the cancer diagnosis.

^c^ Adjusted for sex, age at time of diagnosis (5-year interval), Charlson comorbidity index (0, 1–2, 3+), marital status (married or cohabiting, living alone), education (low, medium, high), and calendar year (2003–2004, 2005–2007, 2008–2010).

^d^ Adjusted for cancer stage at time of diagnosis (local, regional, distant, and unknown) besides the factors included in the analysis ^c^.

^e^ The reference refers to cancer patients who had no AD prescription in the 3 years before cancer diagnosis.
