# Supplementary material for: Cancer Mortality in People Treated with Antidepressants before Cancer Diagnosis: A Population Based Cohort Study
Source: PLoS One. 2015 Sep 14;10(9):e0138134. doi: 10.1371/journal.pone.0138134 (PMC4569483; doi:10.1371/journal.pone.0138134)
Supplement: S1 Table — (DOCX) [file pone.0138134.s005.docx]

| **S1 Table. Stage of cancer at time of diagnosis according to antidepressant (AD) treatment in the three years before cancer diagnosis.** | | | | | | | | | |  |
| --- | --- | --- | --- | --- | --- | --- | --- | --- | --- | --- |
|  | No AD treatment (n=168,551) | | With AD treatment | | | | | | Total | |
|  |  |  | Current user with AD prescription in the 4 months before cancer diagnosis (n=21,851) | | | | Others (n=11,260) | |  |  |
| Stage for specific cancer |  |  | Initiation in the 4 months before cancer diagnosis (n=4,304) | | Initiation in the 5 months or more before cancer diagnosis (n=17,547) | |  |  |  |  |
|  | No. | % | No. | % | No. | % | No. | % | No. | % |
| Bladder cancer |  |  |  |  |  |  |  |  |  |  |
| Localized | 3,562 | 72.9 | 78 | 75.7 | 346 | 79.7 | 187 | 77.0 | 4,173 | 73.6 |
| Regional | 237 | 4.8 | 3 | 2.9 | 15 | 3.5 | 11 | 4.5 | 266 | 4.7 |
| Distant | 381 | 7.8 | 9 | 8.7 | 30 | 6.9 | 17 | 7.0 | 437 | 7.7 |
| Unknown or missing | 707 | 14.5 | 13 | 12.6 | 43 | 9.9 | 28 | 11.5 | 791 | 14.0 |
| Breast |  |  |  |  |  |  |  |  |  |  |
| Localized | 11,025 | 41.3 | 158 | 34.8 | 1,298 | 39.7 | 889 | 43.4 | 13,370 | 41.2 |
| Regional | 5,711 | 21.4 | 84 | 18.5 | 644 | 19.7 | 416 | 20.3 | 6,855 | 21.1 |
| Distant | 947 | 3.6 | 36 | 7.9 | 114 | 3.5 | 71 | 3.5 | 1,168 | 3.6 |
| Unknown or missing | 8,981 | 33.7 | 176 | 38.8 | 1,216 | 37.2 | 672 | 32.8 | 11,045 | 34.0 |
| Colorectal cancer |  |  |  |  |  |  |  |  |  |  |
| Localized | 6,928 | 29.7 | 108 | 25.7 | 673 | 29.8 | 398 | 30.4 | 8,107 | 29.7 |
| Regional | 5,250 | 22.5 | 78 | 18.5 | 426 | 18.9 | 267 | 20.4 | 6,021 | 22.0 |
| Distant | 4,804 | 20.6 | 96 | 22.8 | 441 | 19.5 | 241 | 18.4 | 5,582 | 20.4 |
| Unknown or missing | 6,350 | 27.2 | 139 | 33.0 | 718 | 31.8 | 404 | 30.8 | 7,611 | 27.9 |
| Lung |  |  |  |  |  |  |  |  |  |  |
| Localized | 3,108 | 14.7 | 121 | 12.1 | 513 | 17.2 | 300 | 16.5 | 4,042 | 15.0 |
| Regional | 3,518 | 16.7 | 162 | 16.2 | 462 | 15.5 | 276 | 15.1 | 4,418 | 16.4 |
| Distant | 10,771 | 51.1 | 517 | 51.8 | 1,456 | 48.9 | 914 | 50.1 | 13,658 | 50.8 |
| Unknown or missing | 3,680 | 17.5 | 198 | 19.8 | 545 | 18.3 | 333 | 18.3 | 4,756 | 17.7 |
| Melanoma |  |  |  |  |  |  |  |  |  |  |
| Localized | 6,399 | 70.1 | 70 | 61.9 | 459 | 66.8 | 332 | 68.6 | 7,260 | 69.8 |
| Regional | 627 | 6.9 | 4 | 3.5 | 39 | 5.7 | 35 | 7.2 | 705 | 6.8 |
| Distant | 272 | 3.0 | 4 | 3.5 | 28 | 4.1 | 13 | 2.7 | 317 | 3.0 |
| Unknown or missing | 1,826 | 20.0 | 35 | 31.0 | 161 | 23.4 | 104 | 21.5 | 2,126 | 20.4 |
| Prostate cancer |  |  |  |  |  |  |  |  |  |  |
| Localized | 12,911 | 52.5 | 176 | 40.8 | 706 | 46.4 | 642 | 54.3 | 14,435 | 52.1 |
| Regional | 515 | 2.1 | 11 | 2.6 | 31 | 2.0 | 19 | 1.6 | 576 | 2.1 |
| Distant | 2,865 | 11.7 | 86 | 20.0 | 215 | 14.1 | 140 | 11.8 | 3,306 | 11.9 |
| Unknown or missing | 8,284 | 33.7 | 158 | 36.7 | 568 | 37.4 | 381 | 32.2 | 9,391 | 33.9 |
